# Gold Nanoparticles Inhibit PMA-Induced MMP-9 Expression via microRNA-204-5p Upregulation and Deactivation of NF-κBp65 in Breast Cancer Cells

**DOI:** 10.3390/biology12060777

**Published:** 2023-05-27

**Authors:** Aisha Farhana, Abdullah Alsrhani, Nazia Nazam, Muhammad Ikram Ullah, Yusuf Saleem Khan, Zafar Rasheed

**Affiliations:** 1 Department of Clinical Laboratory Sciences, College of Applied Medical Sciences, Jouf University, Sakaka 72388, Aljouf, Saudi Arabia; 2 Division of Pediatric Surgery, School of Medicine, University of Alabama at Birmingham, Birmingham, AL 35233, USA; 3 Department of Anatomy, College of Medicine, Jouf University, Sakaka 72388, Aljouf, Saudi Arabia; 4 Department of Pathology, College of Medicine, Qassim University, P.O. Box 6655, Buraidah 51452, Qassim, Saudi Arabia

**Keywords:** breast cancer, gold nanoparticles, MMP-9, hsa-miR-204-5p, NF-κBp65, parthenolide

## Abstract

**Simple Summary:**

Breast cancer has a high prevalence in females worldwide, with persistent challenges in its treatment. Gold nanoparticles (AuNPs) have gained popularity for their anti-tumorigenic role, but their therapeutic function in microRNA (miRNA) regulation has never been explored. This study determines the potential of chemically engineered AuNPs against tumorigenic protein matrix metallopeptidase 9 (MMP-9). The data shows that in breast cancer cells, microRNA-204-5p is a direct regulator of MMP-9. AuNPs inhibit MMP-9 expression through the upregulation of microRNA-204-5p in a dose-dependent manner. Moreover, AuNPs also inhibit nuclear transcription factor, NF-κB activation in breast cancer cells transfected with anti-hsa-miR-204. In short, AuNPs are stable and non-toxic to breast cancer cells. AuNPs inhibit MMP-9 expression and NF-κBp65 induction and upregulate microRNA-204. These novel therapeutic potentials of AuNPs on breast cancer cells provide insight that AuNPs inhibit carcinogenic activity via microRNAs.

**Abstract:**

Objective: Breast cancer (BC) is the most common malignancy in females globally. Matrix metalloproteinase-9 (MMP-9) is crucial to the invasion, progression and spread of BC. Gold nanoparticles (AuNPs) have an anti-tumorigenic role, but their therapeutic role in microRNAs (miRNAs) regulation has not been explored. This study determined the potential of AuNPs against MMP-9 overexpression/production and miRNA-204-5p regulation in BC cells. Methods: AuNPs were newly engineered, and their stability was analyzed using the zeta potential, polydispersity index, surface-plasmon-resonance peak and transmission electron microscopy. A bioinformatics algorithm was used to predict the pairing of miRNA in the 3′untranslated-region (3′UTR) of MMP-9 mRNA. TaqMan assays were carried out to quantify miRNA and mRNA, whereas MMP-9-specific immunoassays and gelatin zymography were used to determine protein secretion and activity. The binding of miRNA in MMP-9 mRNA 3′UTR was verified by luciferase reporter clone assays and transfection with anti-miRNAs. In addition, NF-κBp65 activity was determined and confirmed with parthenolide treatment. Results: Engineered AuNPs were highly stable and spherical in shape, with a mean size of 28.3 nm. Tested in MCF-7 BC cells, microRNA-204-5p directly regulates MMP-9. AuNPs inhibit PMA-induced MMP-9 mRNA and protein via hsa-miR-204-5p upregulation. Anti-miR-204 transfected MCF-7 cells demonstrated enhanced MMP-9 expression (*p* < 0.001), while AuNPs treatment attenuated MMP-9 expression in a dose-dependent manner (*p* < 0.05). Moreover, AuNPs also inhibit PMA-induced NF-κBp65 activation in anti-hsa-miR-204 transfected MCF-7 cells. Conclusion: Engineered AuNPs were stable and non-toxic to BC cells. AuNPs inhibit PMA-induced MMP-9 expression, production and activation via NF-κBp65 deactivation and hsa-miR-204-5p upregulation. These novel therapeutic potentials of AuNPs on stimulated BC cells provide novel suggestions that AuNPs inhibit carcinogenic activity via inverse regulation of microRNAs.

## 1. Introduction

Matrix metalloproteinase-9 (MMP-9) portrays a distinct function in the initiation, progression, invasion and tumor microenvironment modulation in several cancers, including colon cancers, lung cancers, brain cancers, melanomas and breast cancer. MMP-9 is highly upregulated in breast cancers, facilitating the infiltration of tumor cells and angiogenesis, mainly through the degradation of the basal membrane. An increase in MMP-9 enhances the metastatic potential of pan-breast cancers [1]. In addition to a prognostic value, MMP-9 is associated with the presence of circulating tumor cells (CTC) during early-stage breast cancer [2]. MMP-9 is induced by a Rho GTPase family protein, namely CDC42, which assists in cytoskeletal organization and ECM adhesion. Elevated CDC42 protein in breast cancer cells modulates epithelial architecture and stimulates the trafficking of matrix metalloproteinase-initiating oncogenes. Low MMP-9 expression in cancer and stromal cells demonstrates a significantly improved disease-free patient survival compared to patients with higher MMP-9 expression [3,4].

Deregulation of microRNAs (miRNA) is another significant feature for cancer initiation, progression and metastasis. Micro RNAs integrate the genomic counterpart of cancer progression into its epigenetic module. It is now apparent that miRNA dysregulation in cancer is not only through epigenomic modulation but miRNAs themselves indirectly program DNA and histone alterations. miRNAs are demonstrated to regulate chromatin remodeling and gene expression patterns. In breast cancer, miRNAs can serve as epigenetic markers and therapeutic targets. miRNAs are shown to affect the epigenetic machinery by binding to 3′UTR in various breast cancers, subsequently reducing the expression of critical enzymes and proteins. miRNA, especially the miR-200 family, has a prominent representation in the epithelial-mesenchymal transition (EMT) of cancer cells. Increased miR221 expression induced mammosphere formation and enriched the CD24−/low/CD44+ stem cell in breast cancers. Additionally, a class of miRNA, termed epi-miRNA, is shown to regulate the stemness of cancer cells in breast cancers [5].

The current increase in breast cancer incidence and deaths have raised concern regarding diagnostic and therapeutic efforts. Numerous research efforts have been channelized, and novel techniques are being discovered and tested. The use of gold nanoparticles has opened a new perspective in theranostics applications against many cancers [6]. Several fields of medicine have benefited from the use of gold nanoparticles. These particles can be effectively and reproducibly synthesized and functionalized, and with the current technologies, gold nanoparticles can be precisely characterized up to the atomic level [7]. In recent years, metal-based nanosystems, including gold-based nanosystems, have been effectively customized to regulate gene expression patterns, molecular and cellular imaging, drug carriers, and photo and thermal-responsive therapeutics. Nanomodulators serve as a safe and effective alternative to molecular-based theranostic systems due to their enhanced and advantageous physicochemical properties [8].

In this study, we hypothesized that engineered AuNPs suppress the tumor-promoting cell signaling events via modulating the microRNA expression. To test this hypothesis, AuNPs were chemically generated, and their suitability for cancer cell treatment was determined by zeta potential, polydispersity index and surface-plasmon-resonance peak. The TargetScan algorithm was used to predict the pairing of miRNA to the 3′untranslated-region (3′UTR) of MMP-9 mRNA. TaqMan assays were used to quantify miRNA and mRNA, whereas MMP-9-specific immunoassays and gelatin zymography were used to determine protein secretion and activity. The binding of miRNA in MMP-9 mRNA 3′UTR was verified by luciferase reporter clone assays and transfection with anti-miRNAs. This is the first report demonstrating that AuNPs inhibit PMA-induced MMP-9 expression and NF-κBp65 activity via upregulation of microRNA-204-5p expression in human BC cells. These findings provide novel suggestions that AuNPs inhibit tumor-promoting activity via modulation of microRNA expression.

## 2. Methods

### 2.1. Chemical Synthesis of Gold (Au) Nanoparticles

The gold nanoparticles (AuNPs) were prepared using the trisodium citrate methods described previously [9,10]. Briefly, the trisodium citrate (Na_3_C_6_H_5_O_7_^.^2H_2_O; 26.2 mM) solution was first prepared in distilled water (catalog #: 15230, Gibco, Life Technologies Corporation, New York city, NY, USA). Simultaneously, chloroauric acid (HAuCl_4_, 2 mM) was added to 10 mL of boiled distilled water. After boiling HAuCl_4_, 4 mL of Na_3_C_6_H_5_O_7_^.^2H_2_O solution (26.2 mM) was added. The final solution of this mixture was boiled for 1 h 30 min and then allowed to cool at RT and centrifuged, and the supernatant was used as AuNPs.

### 2.2. Characterization of AuNPs

Ultraviolet-Visible (UV-Vis) spectrophotometry and dynamic light (DL) scattering (DLS) were used to characterize the chemically synthesized AuNPs. The Lambda XLS Perkin Elmer UV-Vis spectrophotometer (PerkinElmer, Inc., Waltham, MA, USA) was used to measure surface plasmon resonance peak as described previously [11], whereas the particles size, polydispersity index (PDI) and zeta-potential measurements were performed by DLS using Malvern Zetasizer nano 6.01 (Herrenberg, Germany) as previously described [9,10]. Transmission electron microscopy (TEM) was used to analyze the structure of chemically synthesized AuNPs, whereas penetration of AuNPs in breast cancer cells MCF-7 was studied by scanning electron microscopy (SEM). TEM was conducted with Nanopartz, whereas SEM was performed by culturing MCF-7 cells with chemically synthesized AuNPs in cell culture plates containing coverslips, followed by fixation of MCF-7 cells with methanol and dehydration. The coverslips coated with MCF-7 cells and AuNPs were analyzed by SEM using a JEOL JSM-550 scanning electron microscope (Jeol, Akishima, Tokyo, Japan).

### 2.3. Human Adenocarcinoma Breast Cancer Cells MCF-7 Culture

Human BC cell line MCF-7 was obtained from the American Type Culture Collection (Rockville, Rockville, MD, USA) and was culture propagated in DMEM-F12 complete media with 10% heat-inactivated fetal bovine serum (FBS), nonessential amino acids, penicillin (100 U/mL), streptomycin (100 μg/mL) and 10 μg/mL insulin in a 5% CO_2_ incubator at 37 °C as described previously [12].

### 2.4. Treatment of MCF-7 Cells with AuNPs or PMA

MCF-7 cells were cultured in DMEM-F12 complete media and were overnight serum-starved at 70–80% confluency and then treated with AuNPs in several experimental conditions. Viability was examined by Cell Titer-Glo Luminescent Cell Viability Assay kit (catalog # G7573, Promega, Fitchburg, Wl, USA) as described previously [13]. Whereas, in other sets of experiments, serum-starved MCF-7 cells were pretreated with AuNPs (0.2–0.6 µg/mL) for 2 h and subsequently stimulated with 0.1 PMA (catalog # P8139, Sigma–Aldrich, St. Louis, MO, USA) for 24 h, whereas the MCF-7 cells cultured without AuNPs or PMA served as a negative control.

### 2.5. Target Scan Bioinformatics Approach

Target Scan bioinformatics approach (http://www.targetscan.org/, accessed on 11 January 2023) was used for the analysis of microRNAs complementary to 3′UTR of mRNA for MMP-9. The approach for the prediction of miRNAs sequences in the 3′UTR of pathogenic genes was the same as described previously [14]. 

### 2.6. Transfection of MCF-7 Cells with Anti-miRNAs

MCF-7 cell transfection was carried out with miRNA inhibitors (anti-miRNAs; 100 nM; Ambion/Qiagen). HiPerfect Transfection Reagent was used according to the manufacturer’s instructions (Qiagen, Germantown, MD, USA). After 72 h post-transfection, MCF-7 cells were treated with 0.2–0.6 µg/mL of AuNPs for 2 h or stimulated with PMA (0.1 µM) for 24 h, and cell lysate or RNA samples were prepared for further analysis.

### 2.7. Luciferase Reporter Assays

The miRNA pairing in the 3′ untranslated region (′UTR) of mRNA pathogenic genes was verified by luciferase reporter assays (Promega, WI, USA) [15]. Briefly, a luciferase reporter vector having the complete 3′UTR of MMP-9 mRNA (# ENST00000372330.3) and an empty vector exclusively containing luciferase gene and active promoter (Applied Biological Materials Inc., BC, Canada, or SwitchGear Genomics, Menlo Park, CA, USA) were used in the reporter assays. MCF-7 cells were co-transfected with a reporter plasmid (100 nM), miRNA inhibitor (50–100 nM), or control miRNAs (negative control) using HiPerfect Transfection Reagent (Qiagen, Germantown, MD, USA). After 72 h of transfection, MCF-7 cells were treated with AuNPs (0.2–0.4 µg/mL), and dual luciferase activity was determined according to the manufacturer procedure (Promega Corporation, Fitchburg, WI, USA).

### 2.8. Quantitative Real-Time PCR for miRNA and mRNA Analysis

Total RNA harboring miRNA fraction was prepared from the treated and untreated MCF-7 cells by mirVana miRNA isolation kit as directed by the manufacturer’s instructions (Ambion, Foster City, CA, USA). The Superscript First Strand cDNA synthesis kit was used for the synthesis of first-strand cDNAs from total RNA following the manufacturer’s instructions (Applied Biosystems, Carlsbad, CA, USA). Expression of mRNAs or miRNAs was quantified by their specific TaqMan assays (Applied Biosystems) using StepOne real-time PCR system (Life Technologies, Foster City, CA, USA). The primer sequences for human MMP-9 mRNA (NM_004994) used were forward 5′GCCACTACTGTGCCTTTGAGTC-3′ and reverse 5′-CCCTCAGAGAATCGCCAGTACT-3′. The primers sequence for human GAPDH (NM_001256799) used were forward 5′GTCTCCTCTGACTTCAACAGCG3′ and reverse 5′ ACCACCCTGTTGCTGTAGCCAA3′. The relative gene expression of the desired mRNA and miRNA was quantified using the double delta CT method described previously [16]. 

### 2.9. Gelatin Zymography

Gelatin zymography was carried out following the published procedure as described previously [17]. An equal volume of the culture medium from treated and non-treated MCF-7 cells was assimilated with non-reducing sample buffer (4% SDS, 0.15 M Tris; pH 6.8) and 20% glycerol containing 0.05% bromophenol blue and resolved on a 10% polyacrylamide gel containing copolymerized 0.2% gelatin (Bio-Rad, Hercules, CA, USA). Human MMP-9 transfected 293T lysate was also run on the same SDS-PAGE and was used as MMP-9 positive control. After completion of the gel electrophoresis, the resolved gels were washed with 2.5% Triton X-100 twice, each time for 15 min, and were incubated in the digestion gelatinase buffer (50 mM Tris-HCl (pH 7.6), 10 mM CaCl_2_, 50 mM NaCl and 0.05% Brij-35) at 37 °C for 16 h. Coomassie brilliant blue R350 staining was used to determine the locations of gelatinolytic activity. Electrophoretic migration of MMP-9 was compared with known molecular weight standards (Catalog # 161–0399, Bio-Rad, Hercules, CA, USA) and clear bands of MMP-9 activity produced by Human MMP-9 transfected 293T lysate. Band images of zymographic gel were digitally taken, and the band intensities (pixels/band) were analyzed using the Un-Scan-It software (Silk Scientific Corporation, Orem, UT, USA) and were represented in average pixels.

### 2.10. Nuclear Factor (NF)-Kappa B p65 Assay

Activated transcription factor NF-κBp65 in nuclear extracts of treated or untreated MCF-7 cells were determined using a Transcription Factor Immunoassay Kit following the manufacturer’s instructions (catalog # ab133128, Abcam, Waltham, Boston, USA). An experimental control, the NF-κBp65 combo positive control (Abcam), was also used. The activated NF-κBp65 in the reaction mixture was quantified at 450 nm using a microplate reader (Anthos 3100, Salzburg, Austria). The nuclear extracts used in the assays were prepared by a published procedure [18]. 

## 3. Results

### 3.1. Characterization of Gold Nanoparticles Generated by Trisodium Citrate Methods

**Figure 1:**
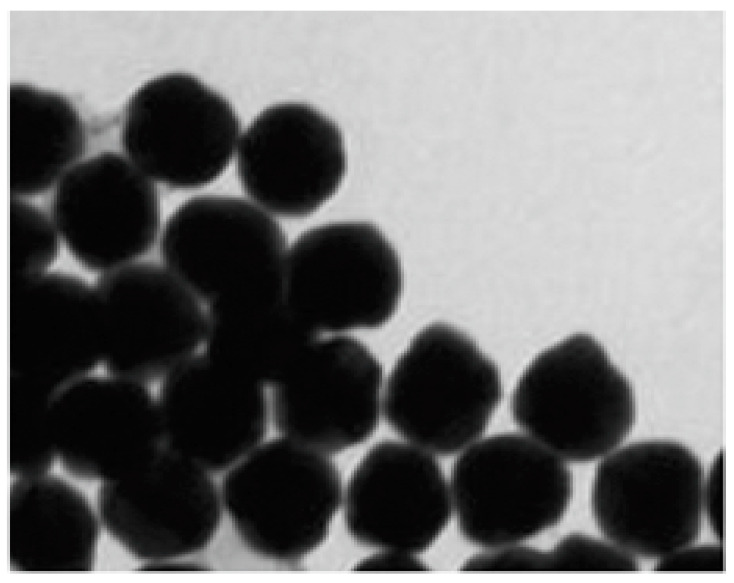
TEM image of chemically synthesized gold nanoparticles (AuNPs).

Dynamic light scattering, UV-Vis spectrophotometry and TEM were used for the characterization of the newly generated AuNPs. Using dynamic light scattering, the average size of AuNPs was quantified to be 28.3 nm, whereas zeta potential was measured to be −32.2 mV, and the polydispersity index of the AuNPs was determined to be 0.435. The surface plasmon resonance peak was analyzed at 524 nm by UV-vis spectrophotometry. In addition, the shape of AuNPs was also analyzed by TEM. The TEM results in Figure 1 showed that the overall shape of AuNPs was spherical. These parameters indicated that chemically synthesized AuNPs are highly stable. The complete details of AuNPs are summarized in Table 1. 

### 3.2. Viability of MCF-7 Breast Cancer Cells Treated with AuNPs

**Figure 2:**
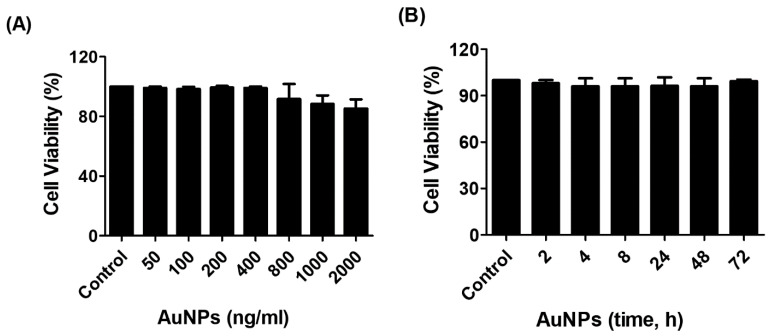
The outcome of gold nanoparticles (AuNPs) on the viability of human breast cancer cell line MCF-7. (**A**) Percent viability of MCF-7 cells against AuNPs treatment. The MCF-7 cells were treated with 50–2000 ng/mL of AuNPs for 24 h. (**B**) Percent viability of MCF-7 cells against indicative treatment time of AuNPs. The MCF-7 cells were treated with 400 ng/mL of AuNPs for 2–72 h. The AuNPs treated MCF-7 breast cancer cells were incubated in serum-starved DMEM culture medium at 37 °C in 5% CO_2,_ and the percent viability was determined by the Cell Titer-Glo Luminescent Cell Viability Assay kit (Promega Corporation, Fitchburg, WI, USA). The data shown are mean ± SD of three independent experiments.

The cytotoxicity of AuNPs was determined by the Cell Titer-Glo Luminescent Cell Viability Assay kit (Promega Corporation, Fitchburg, WI, USA) upon treatment of MCF-7 cells, as described in the methods section. As shown in Figure 2A, the percent cell viability of MCF-7 cells was very stable up to the treatment with 400 ng/mL AuNPs for 24 h and then slightly declined on the further increase of AuNPs concentration. We also determined the viability of MCF-7 cells against treatment time, and the results are presented in Figure 2B. Treatment of MCF-7 cells with 0.4 µg/mL of AuNPs for 0–72 h in serum-starved MCF-7 cells was found to be non-toxic. Based on these findings, the AuNPs used in further studies were 0.2–0.6 µg/mL.

### 3.3. SEM Analysis of AuNPs Penetration in MCF-7 Cells

**Figure 3:**
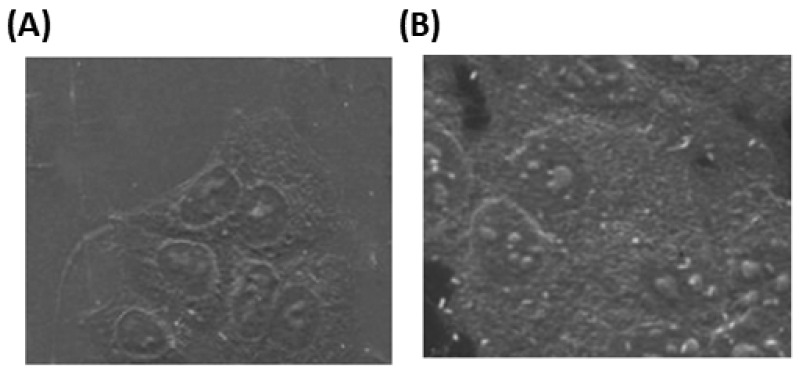
Scanning electron microscopic (SEM) image of gold nanoparticles (AuNPs) in MCF-7 cells. The MCF-7 cells were incubated with 0.4 µg/mL of AuNPs for 2 h (**A**) and 24 h (**B**) in a culture plate containing coverslip in DMEM at 37 °C in a 5% CO_2_ incubator, followed by fixation and dehydration.

The intake of AuNPs by MCF-7 cells was visualized by SEM imaging. SEM studies showed that after 2 h incubation of 0.4 µg/mL of AuNPs with MCF-7 cells, the AuNPs were found in the cancer cells (Figure 3A), and this accumulation of AuNPs was markedly higher after 24 h insulation (Figure 3B), indicating that intake of AuNPs by MCF-7 cells was time-dependent.

### 3.4. Bioinformatic Determination of Binding of hsa-miR-204-5p in 3′UTR of Human MMP-9 mRNA (NM_004994)

**Figure 4:**
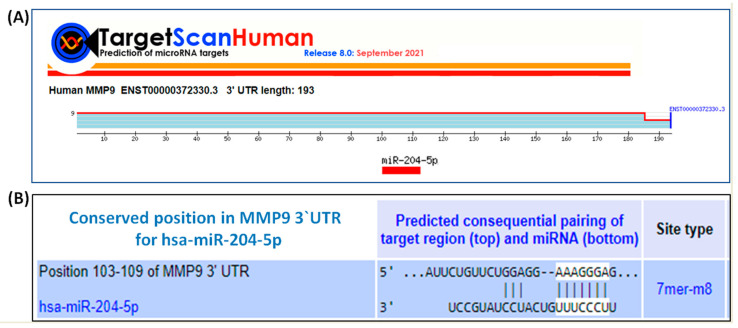
TargetScan bioinformatic algorithm for the analysis of the seed-matched sequence of hsa-miR-204-5p in 3′UTR of human MMP-9. (**A**) Human MMP-9 mRNA 3′UTR (ENST00000372330.3) predicts conserved site of hsa-miR-204-5p at location 103–109 of MMP-9 3′UTR. (**B**) Duplex of hsa-miR-204-5p with the seed-matched sequence in the 3′UTR of human MMP-9 mRNA. The sequences shown in the orange rectangle are the potential locations for the formation of miRNA-mRNA duplex.

TargetScan bioinformatic algorithm was used to predict the complementary sequence between hsa-miR-204-5p and 3′UTR of human MMP-9 mRNA (ENST00000372330.3). TargetScan algorithm shows that human MMP-9 mRNA has 193 nucleotide bases (Figure 4A) and has a conserved site for hsa-miR-204-5p at position 103–109 nucleotide bases (Figure 4B). Furthermore, the predicted consequential pairing between 3′UTR of human MMP-9 mRNA and hsa-miR-204-5p is shown in Figure 4B. 

### 3.5. Experimental Validation of Bioinformatically Predicted Pairing of hsa-miR-204-5p with Human 3′UTR MMP-9 mRNA

**Figure 5:**
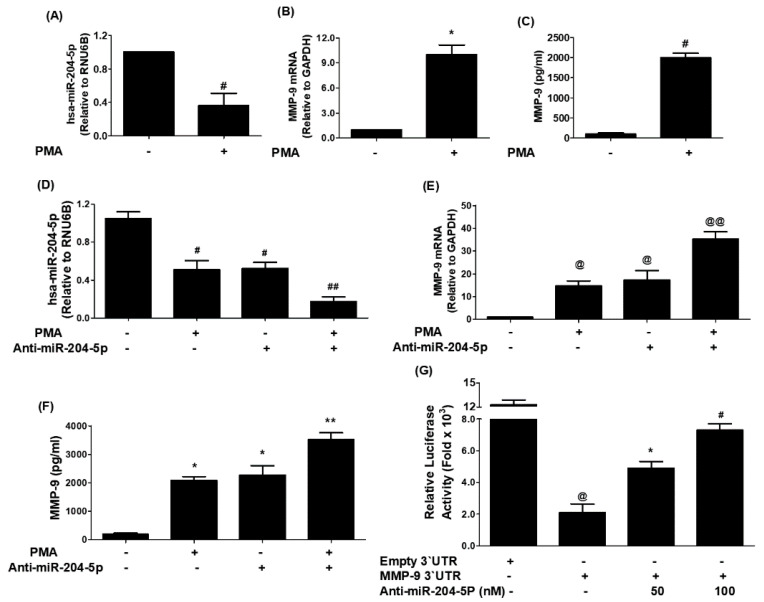
Negative co-relation of hsa-miR-204-5p with MMP-9 expression. (**A**) Expression of hsa-miR-204-5p in Phorbol myristate acetate (PMA)-stimulated MCF-7 human breast cancer cells determined by TaqMan assays. Unstimulated MCF-7 cells were used as controls, and expression of RNU6B was used as an endogenous control. ^#^ *p* < 0.01 versus untreated MCF-7 cells. (**B**) PMA-induced expression of MMP-9 mRNA in MCF-7 cells determined by TaqMan assays. Untreated MCF-7 cells were used as controls, and expression of GAPDH was used as an endogenous control. * *p* < 0.001 versus untreated MCF-7 cells. (**C**) PMA-induced production of MMP-9 in the culture medium of MCF-7 cells determined by human MMP-9 immunoassays. A culture medium obtained from unstimulated MCF-7 cells was used as a control. ^#^ *p* < 0.01 versus untreated MCF-7 cells. (**D**) PMA-suppressed expression of hsa-miR-204-5p in anti-miR-204-5p transfected MCF-7 cells determined by TaqMan assays. Unstimulated MCF-7 cells were used as controls, and expression of RNU6B was used as an endogenous control. ^#^ *p* < 0.05 versus untreated MCF-7 cells; ^#^ *p* < 0.05 versus ^##^. (**E**) PMA-induced expression of MMP-9 mRNA in anti-miR-204-5p transfected MCF-7 cells determined by TaqMan assays. Unstimulated MCF-7 cells were used as controls, and expression of RNU6B was used as an endogenous control. ^@^ *p* < 0.05 versus untreated MCF-7 cells; ^@^ *p* < 0.05 versus ^@@^. (**F**) PMA-induced production of MMP-9 in the culture medium of anti-miR-204-5p transfected MCF-7 cells determined by human MMP-9 immunoassays. A culture medium obtained from unstimulated MCF-7 cells was used as a control. * *p* < 0.01 versus untreated MCF-7 cells; * *p* < 0.05 versus **. (**G**) Luciferase activity in MCF-7 cells transfected with the reporter vector containing the entire 3′UTR of MMP-9 mRNA (MMP-9 3′UTR) and anti-miR-204-5p. Transfection of MCF-7 with an empty 3′UTR vector (vector containing only luciferase gene and active promoter) alone and MMP-9 3′UTR alone was used as a negative and positive control, respectively. ^@^ *p* < 0.001 versus MCF-7 cells transfected with empty 3′UTR; * *p* < 0.05 versus @; ^#^ *p* < 0.01 versus ^@^. The data shown are mean ± SD of three independent experiments.

Stimulation of MCF-7 cells with PMA significantly downregulated hsa-miR-204-5p expression (Figure 5A; *p* < 0.05) and significantly upregulated the mRNA expression of human MMP-9 (Figure 5B; *p* < 0.001). The effect of PMA stimulation has also significantly increased the protein production of MMP-9 in the culture medium (Figure 5C; *p* < 0.001). These results were further validated in another set of MCF-7 cells transfected with anti-miR-204-5p and then stimulated with PMA. As shown in Figure 5D, treatment of anti-miR-204-5p-transfected MCF-7 cells with PMA significantly inhibited the expression of miR-204-5p expression as compared with those sets of anti-miR-204-5p-transfected MCF-7 cells without PMA treatment (*p* < 0.05), and these results were the same when compared with those sets of MCF-7 cells which were non-transfected MCF-7 but stimulated with PMA alone (*p* < 0.05). Interestingly, these results were completely reversed when we estimated the MMP-9 at mRNA (Figure 5E) and protein levels (Figure 5F). Treatment of anti-miR-204-5p-transfected MCF-7 cells with PMA significantly increased the expression of MMP-9 mRNA as compared with those sets of anti-miR-204-5p-transfected MCF-7 cells without PMA treatment (*p* < 0.05), and with those sets of MCF-7 cells which were non-transfected MCF-7 but stimulated with PMA alone (*p* < 0.05). These results confirmed the inverse correlation between miR-204-5p and MMP-9 expression in MCF-7 cells and that miR-204-5p is a direct regulator of MMP-9 expression at mRNA (Figure 5F) and protein (Figure 5F). To confirm whether this inverse regulation between miR-204-5p and MMP-9 is involved in 3′UTR of MMP-9 mRNA, we used the reported luciferase assay using co-transfection of MCF-7 cells with a reporter clone containing entire 3′UTR of MMP-9 (MMP-9 3′UTR) and miR-204-5p inhibitor (anti-miR-204-5p). A significant increase in luciferase activity was observed in MCF-7 cells co-transfected with MMP-9 3′UTR and anti-miR-204-5p as compared with those MCF-7 cells transfected with MMP-9 3′UTR alone (*p* < 0.05). The transfection of MCF-7 cells with MMP-9 3′UTR alone significantly reduced the relative luciferase activity as compared with those MCF-7 cells transfected with empty 3′UTR (*p* < 0.0001) (Figure 5G). These results confirmed the role of 3′UTR of human MMP-9 mRNA in the regulation of hsa-miR-204-5p activity.

### 3.6. AuNPs Upregulate hsa-miR-204-5p Expression and Inhibit MMP-9 mRNA Expression and Protein Production

**Figure 6:**
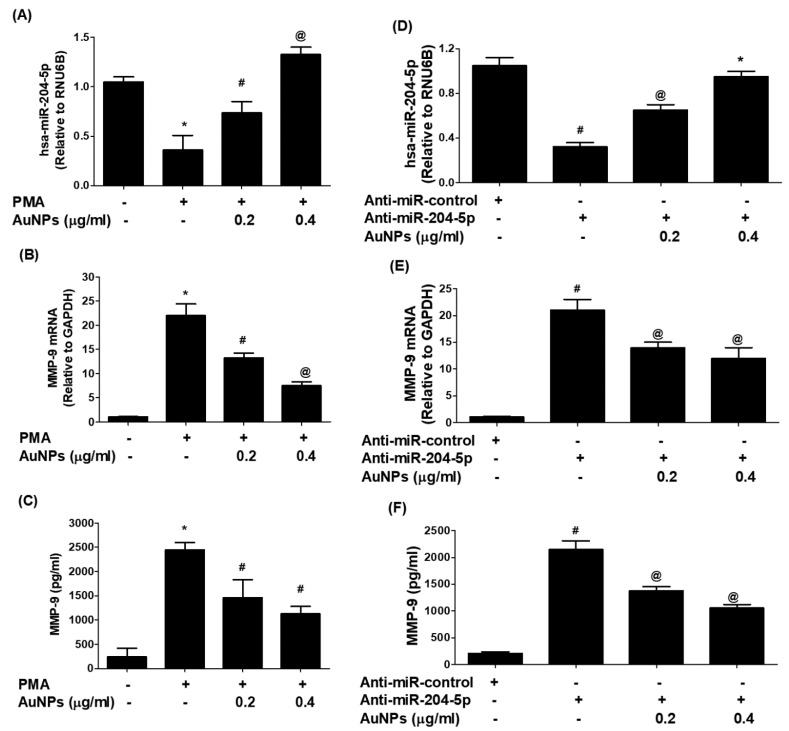
Gold nanoparticles (AuNPs) modulated expression of micrRNA-204-5p and MMP-9 in MCF-7 breast cancer cells. (**A**) AuNPs upregulate the expression of hsa-miR-204-5p in PMA-stimulated MCF-7 breast cancer cells. MCF-7 cells were pretreated with AuNPs (0.2–0.4 μg/mL) for 2 h and stimulated by PMA (0.1 µM) for 24 h. Unstimulated MCF-7 cells were used as an experimental negative control. Comparative expression of hsa-miR-204-5p was determined by TaqMan assays using RNU6B expression as an endogenous control. * *p* < 0.05 versus unstimulated MCF-7 cells; ^#^ *p* < 0.05 versus *; ^@^ *p* < 0.01 versus *; ^@^ *p* < 0.05 versus ^#^. (**B**) AuNPs inhibit the expression of MMP-9 mRNA in PMA-stimulated MCF-7 breast cancer cells. MCF-7 cells were pretreated with AuNPs (0.2–0.4 μg/mL) for 2 h and stimulated by PMA (0.1 µM) for 24 h. Unstimulated MCF-7 cells were used as an experimental negative control. Comparative expression of MMP-9 mRNA was determined by TaqMan assays using GAPDH expression as an endogenous control. * *p* < 0.001 versus unstimulated MCF-7 cells; ^#^ *p* < 0.05 versus *; ^@^ *p* < 0.01 versus *; ^@^ *p* < 0.05 versus ^#^. (**C**) AuNPs inhibit MMP-9 protein production in the culture medium of PMA-stimulated MCF-7 breast cancer cells. MCF-7 cells were pretreated with AuNPs (0.2–0.4 μg/mL) for 2 h and stimulated by PMA (0.1 µM) for 24 h, and the levels of MMP-9 in the culture medium were determined by human MMP-9 immunoassays. A culture medium from unstimulated MCF-7 cells was used as an experimental negative control. * *p* < 0.0001 versus unstimulated MCF-7 cells; ^#^ *p* < 0.05 versus *; ^@^ *p* < 0.01 versus *; ^@^ *p* < 0.05 versus ^#^. (**D**) AuNPs upregulate the expression of hsa-miR-204-5p in MCF-7 cells transected with anti-miR-204-5p. Anti-miR-204-5p transfected MCF-7 cells were incubated with AuNPs (0.2–0.4 μg/mL) for 24 h. MCF-7 cells transfected with anti-miR-control were used as an experimental negative control. Comparative expression of hsa-miR-204-5p was determined by TaqMan assays using RNU6B expression as an endogenous control. ^#^*p* < 0.01 versus anti-miR-control transfected MCF-7 cells; ^#^ *p* < 0.05 versus ^@^; ^@^ *p* < 0.01 versus *; * *p* < 0.01 versus ^#^. (**E**) AuNPs inhibit the expression of MMP-9 mRNA in MCF-7 cells transected with anti-miR-204-5p. Anti-miR-204-5p transfected MCF-7 cells were incubated with AuNPs (0.2–0.4 μg/mL) for 24 h. MCF-7 cells transfected with anti-miR-control were used as an experimental negative control. Comparative expression of MMP-9 mRNA was determined by TaqMan assays using GAPDH expression as an endogenous control. ^#^ *p* < 0.001 versus anti-miR-control transfected MCF-7 cells; ^#^ *p* < 0.05 versus ^@^. (**F**) AuNPs inhibit MMP-9 protein production in MCF-7 cells transected with anti-miR-204-5p. Anti-miR-204-5p transfected MCF-7 cells were incubated with AuNPs (0.2–0.4 μg/mL) for 24 h. MCF-7 cells transfected with anti-miR-control were used as an experimental negative control. MMP-9 levels in the culture medium were determined by human MMP-9 immunoassays. ^#^ *p* < 0.001 versus anti-miR-control transfected MCF-7 cells; ^#^ *p* < 0.05 versus ^@^. The data shown are mean ± SD of three independent experiments.

The therapeutic effect of AuNPs against the potent proinflammatory mediator MMP-9 was examined on the cancer cells treated by PMA. As shown in Figure 6A, treatment of MCF-7 cells with PMA alone significantly downregulates the expression of hsa-miR-204-5p, whereas treatment of the same set of MCF-7 cells with AuNPs prior to the treatment with PMA significantly upregulated the expression of miR-204-5p. Interestingly, this condition was reversed when we estimated the levels of MMP-9 mRNA and protein in the same sets of MCF-7. Treatment of MCF-7 cells with PMA alone significantly upregulates the MMP-9 mRNA expression, whereas treatment of the same set of MCF-7 cells with AuNPs before the treatment with PMA significantly inhibited the expression of MMP-9 mRNA in a dose-dependent manner (Figure 6B; *p* < 0.05), and MMP-9 secretion in the culture medium (Figure 6C; *p* < 0.05). To re-validate the therapeutic effects of AuNPs on MMP-9 expression via hsa-miR-204-5p, the MCF-7 cells were transfected with anti-miR-204-5p and then treated with AuNPs after 72 h post-transfection. Transfection of MCF-7 cells with anti-miR-204-5p significantly downregulates the expression of hsa-miR-204-5p, whereas treatment of anti-miR-204-5p-transfected-MCF-7 cells with AuNPs significantly upregulated the expression of miR-204-5p in a dose-dependent manner (Figure 6D). Interestingly, this condition was reversed when we estimated the levels of MMP-9 mRNA (Figure 6E) and MMP-9 secretion (Figure 6F) in the same sets of anti-miR-204-5p-transfected MCF-7. Transfection of MCF-7 cells with anti-miR-204-5p significantly upregulates the expression of MMP-9 mRNA (*p* < 0.01), whereas treatment of anti-miR-204-5p-transfected-MCF-7 cells with AuNPs significantly inhibited MMP-9 mRNA expression (*p* < 0.05) and also significantly inhibited MMP-9 protein secretion in a dose-dependent manner (*p* < 0.05).

### 3.7. AuNPs Inhibit MMP-9 Expression via Upregulation of hsa-miR-204-5p through 3′UTR of MMP-9 mRNA in MCF-7 Cells

**Figure 7:**
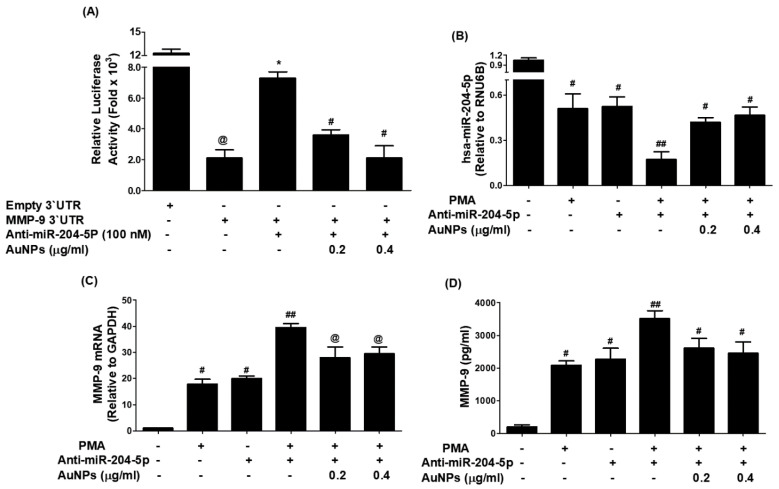
Gold nanoparticles (AuNPs) inhibit MMP-9 expression via upregulation of hsa-miR204-5p in MCF-7 cells. (**A**) Effect of AuNPs on the luciferase activity in MCF-7 cells co-transfected with MMP-9 3′UTR reporter vector and anti-miR-204-5p. Transfection of MCF-7 cells with Empty 3′UTR vector alone and MMP-9 3′UTR alone was used as negative and positive controls, respectively. ^@^ *p* < 0.0001 vs. MCF-7 cells transfected with an empty 3′UTR vector; * *p* < 0.001 vs. ^@^; * *p* < 0.05 vs. ^#^. (**B**) AuNPs upregulate hsa-miR-204-5p expression in anti-miR-204-5p-transfected MCF-7 cells stimulated with PMA. Anti-miR-204-5p transfected MCF-7 cells were pretreated with AuNPs (0.2–0.4 μg/mL) for 2 h and stimulated by PMA (0.1 µM) for 24 h. Unstimulated/non-transfected MCF-7 cells were used as an experimental negative control. Comparative expression of hsa-miR-204-5p was determined by TaqMan assays using RNU6B expression as an endogenous control. ^#^ *p* < 0.01 versus non-transfected MCF-7 cells; ^#^ *p* < 0.05 versus ^##^. (**C**) AuNPs inhibit MMP-9 mRNA expression in anti-miR-204-5p-transfected MCF-7 cells stimulated with PMA. Anti-miR-204-5p transfected MCF-7 cells were pretreated with AuNPs (0.2–0.4 μg/mL) for 2 h and stimulated by PMA (0.1 µM) for 24 h. Unstimulated/non-transfected MCF-7 cells were used as an experimental negative control. Comparative expression of MMP-9 mRNA was determined by TaqMan assays using GAPDH expression as an endogenous control. ^#^ *p* < 0.05 versus non-transfected MCF-7 cells; ^##^ *p* < 0.05 versus ^@^. (**D**) AuNPs inhibit MMP-9 production in the culture medium of anti-miR-204-5p-transfected MCF-7 cells stimulated with PMA. Anti-miR-204-5p transfected MCF-7 cells were pretreated with AuNPs (0.2–0.4 μg/mL) for 2 h and stimulated by PMA (0.1 µM) for 24 h. The culture medium obtained from unstimulated/non-transfected MCF-7 cells was used as an experimental negative control. MMP-9 production levels in the culture medium were determined by MMP-9-specific immunoassays. ^#^ *p* < 0.05 versus non-transfected MCF-7 cells; ^##^ *p* < 0.05 versus ^#^. The data shown are mean ± SD of three independent experiments.

**Figure 8:**
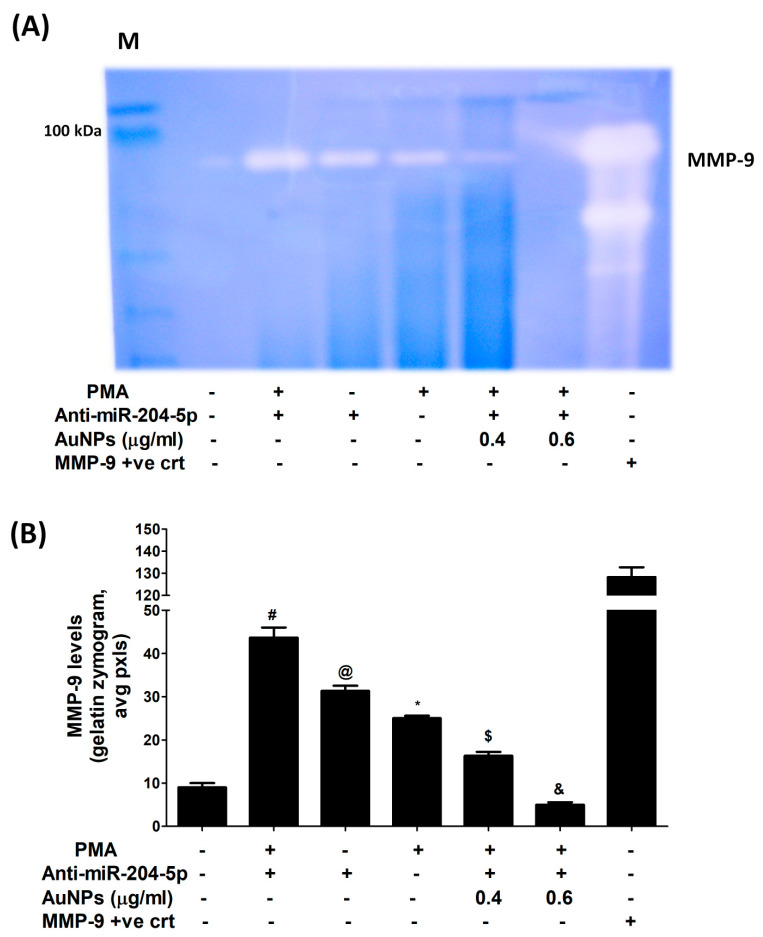
Gelatin zymographic-based MMP-9 activity in the culture medium of anti-miR-204-5p-transfeced MCF-7 cells treated with AuNPs and PMA. (**A**) Anti-miR-204-5p transfected MCF-7 cells were pretreated with AuNPs (0.4–0.6 μg/mL) for 2 h and stimulated by PMA (0.1 µM) for 24 h, and equal volumes of culture medium were loaded on a polyacrylamide gel and MMP-9 activity was analyzed by gelatin zymography. A culture medium obtained from unstimulated/non-transfected MCF-7 cells was used as an experimental negative control. Human MMP-9 transfected 293T lysate was used as MMP-9 positive control. (**B**) Band images of zymographic gel were digitally captured, and the band intensities (pixels/band) were obtained using the Un-Scan-It software and were expressed in average pixels. ^#^ *p* < 0.001 versus unstimulated/non-transfected MCF-7 cells; ^@^ *p* < 0.05 versus ^#^; * *p* < 0.05 versus ^#^; ^$^ *p* < 0.01 versus ^#^; ^&^ *p* < 0.001 versus ^#^.

Attenuation of MMP-9 expression by AuNPs via involvement of 3′UTR of MMP-9 and hsa-miR-204-5p was validated by luciferase reporter assays. As shown in Figure 7A, a significant increase in the relative luciferase activity was observed in MCF-7 cells, co-transfected with MMP-9 3′UTR reporter and anti-miR-204-5p (*p* < 0.001). This increase in luciferase activity was inhibited by the treatment of these co-transfected MCF-7 cells with AuNPs in a dose-dependent manner (*p* < 0.05). These results indicated that AuNPs inhibit MMP-9 expression via upregulation of hsa-miR-204-5p through 3′UTR of MMP-9 mRNA in MCF-7 cells. The therapeutic potential of AuNPs against MMP-9 expression via hsa-miR-204-5p was further re-evaluated by transfection with anti-miR-204-5p and then treated with PMA and AuNPs. Treatment of anti-miR155-transfected MCF-7 cells with PMA significantly inhibited the expression of hsa-miR-204-5p compared to non-transfected MCF-7 cells stimulated with PMA (*p* < 0.05). Interestingly, treatment with AuNPs significantly upregulated PMA-inhibited hsa-miR-204-5p expression in a dose-dependent manner (Figure 7B; *p* < 0.05). Surprisingly, this condition was reversed when we estimated MMP-9 mRNA and MMP-9 protein secretion. Stimulation of anti-miR-204-5p-transfected MCF-7 cells with PMA significantly enhanced the expression of MMP-9 mRNA as compared with non-transfected MCF-7 cells stimulated with PMA (*p* < 0.01). Interestingly, treatment with AuNPs significantly inhibited PMA-induced MMP-9 mRNA expression in a dose-dependent manner (Figure 7C; *p* < 0.05). To determine whether this inhibition of MMP-9 mRNA expression also affected the protein level, the cell culture medium of these transfected or non-transfected MCF-7 cells was analyzed. The data showed that treatment with AuNPs significantly inhibited PMA-induced MMP-9 protein secretion in a dose-dependent manner (Figure 7D; *p* < 0.05). These results were further validated by gelatin zymography based MMP-9 activity in the culture medium of anti-miR-204-5p-transfected MCF-7 cells treated with AuNPs and PMA. Anti-miR-204-5p transfected MCF-7 cells were pretreated with AuNPs (0.4–0.6 μg/mL) for 2 h and stimulated by PMA (0.1 µM) for 24 h, and equal volumes of culture medium were loaded on a polyacrylamide gel and MMP-9 activity was analyzed by gelatin zymography. The results shown in Figure 8A,B showed that treatment with AuNPs significantly inhibited PMA-induced MMP-9 activity in a dose-dependent manner (*p* < 0.05). In sum, the results indicate that AuNPs suppressed MMP-9 mRNA expression and MMP-9 secretion via upregulation of hsa-miR-204-5p expression.

### 3.8. AuNPs Inhibit PMA-Induced NF-κBp65 Activation via hsa-miR-204-5p in MCF-7 Cells

**Figure 9:**
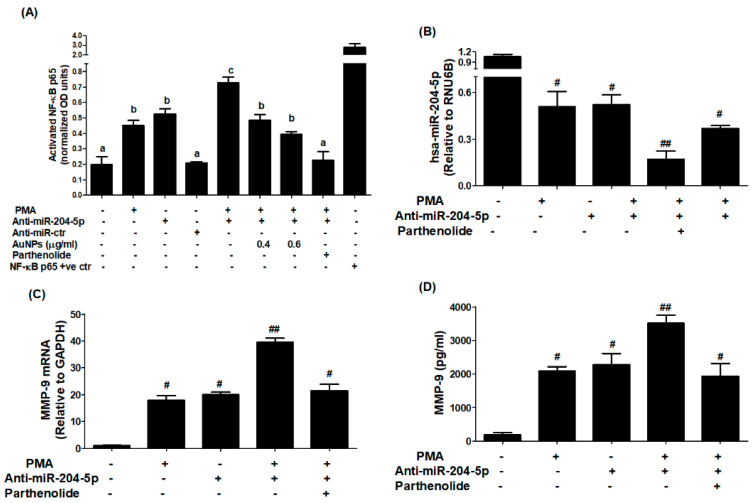
AuNPs inhibit PMA-induced transcription factor NF-κB activation via hsa-miR-204-5p in MCF-7 cells transfected with anti-miR-204-5p. (**A**) AuNPs inhibit PMA-induced NF-κBp65 activation in MCF-7 cells transfected with anti-miR-204-5p determined by NF-κBp65 transcription factor kit (Abcam, Waltham, Boston, USA). The NF-κBp65 positive control was supplied with the kit. Transfection of MCF-7 cells with anti-miR-ctr was used as a negative control, whereas treatment of MCF-7 cells with parthenolide (a marker of NF-κB inhibitor) was used as a positive control of the experiment. The data shown are mean ± SD of three independent experiments. The data differ without a common letters, *p* < 0.05. (**B**) Parthenolide upregulates hsa-miR-204-5p expression in anti-miR-204-5p-transfected MCF-7 cells stimulated with PMA. Anti-miR-204-5p transfected MCF-7 cells were pretreated with parthenolide for 2 h and stimulated by PMA (0.1 µM) for 24 h. Unstimulated/non-transfected MCF-7 cells were used as an experimental negative control. Comparative expression of hsa-miR-204-5p was determined by TaqMan assays using RNU6B expression as an endogenous control. ^#^ *p* < 0.01 versus non-transfected MCF-7 cells; ^#^ *p* < 0.05 versus ^##^. (**C**) Parthenolide inhibits MMP-9 mRNA expression in anti-miR-204-5p-transfected MCF-7 cells stimulated with PMA. Anti-miR-204-5p transfected MCF-7 cells were pretreated with parthenolide for 2 h and stimulated by PMA (0.1 µM) for 24 h. Unstimulated/non-transfected MCF-7 cells were used as an experimental negative control. Comparative expression of MMP-9 mRNA was determined by TaqMan assays using GAPDH expression as an endogenous control. ^#^ *p* < 0.05 versus non-transfected MCF-7 cells; ^##^ *p* < 0.05 versus ^#^. (**D**) Parthenolide inhibits MMP-9 production in the culture medium of anti-miR-204-5p-transfected MCF-7 cells stimulated with PMA. Anti-miR-204-5p transfected MCF-7 cells were pretreated with parthenolide for 2 h and stimulated by PMA (0.1 µM) for 24 h. The culture medium obtained from unstimulated/non-transfected MCF-7 cells was used as an experimental negative control. MMP-9 production levels in the culture medium were determined by MMP-9-specific immunoassays. ^#^ *p* < 0.01 versus non-transfected MCF-7 cells; ^##^ *p* < 0.05 versus ^#^. The data shown are mean ± SD of three independent experiments.

Inhibition of PMA-induced NF-κBp65 activation by AuNPs via involvement of MMP-9 and hsa-miR-204-5p was studied by transfection of MCF-7 cells with anti-miR-204-5p. The results in Figure 9A showed a significant increase of NF-κBp65 levels in the nuclear extract of MCF-7 cells treated with PMA alone for 30 min compared to untreated MCF-7 cells (*p* < 0.01). Similarly, a significant increase of NF-κB p65 levels in the nuclear extract was also observed in MCF-7 cells transfected with anti-miR-204-5p compared to those MCF-7 cells transfected with anti-miR-control (*p* < 0.01). Interestingly, a sharp increase in the levels of NF-κBp65 was observed when transfected MCF-7 cells with anti-mR-204-5p were stimulated with PMA (*p* < 0.001). Importantly, this increase of NF-κBp65 was significantly inhibited by AuNPs in a dose-dependent manner (*p* < 0.05). Treatment of anti-miR-204-5p-transfected MCF-7 cells with parthenolide (a marker of NF-κB inhibitor) showed a significantly inhibited PMA-induced NF-κBp65 activation (Figure 9A). To confirm the involvement of NF-κBp65 inhibition by parthenolide via modulation of hsa-miR-204-5p and MMP-9 expression. The effect of parthenolide was tested on anti-miR-204-5p-transfected MCF-7 stimulated PMA. Treatment of anti-miR-204-5p-transfected MCF-7 cells with parthenolide significantly upregulated PMA-inhibited hsa-miR-204-5p expression in a dose-dependent manner (Figure 9B; *p* < 0.05). This condition was reversed when we estimated MMP-9 mRNA and MMP-9 protein secretion. Treatment of anti-miR-204-5p-transfected MCF-7 cells with parthenolide significantly inhibited PMA-induced MMP-9 mRNA expression (Figure 9C; *p* < 0.05) and significantly inhibited MMP-9 production in the culture medium (Figure 9D; *p* < 0.05), suggesting that AuNPs inhibited MMP-9 expression via hsa-miR-204-5p upregulation and NF-κB deactivation. Altogether, these results confirmed that the chemically engineered AuNPs inhibit MMP-9 expression via hsa-miR-204-5p upregulation and NF-κBp65 deactivation in human breast cancer cells. 

## 4. Discussion

**Figure 10:**
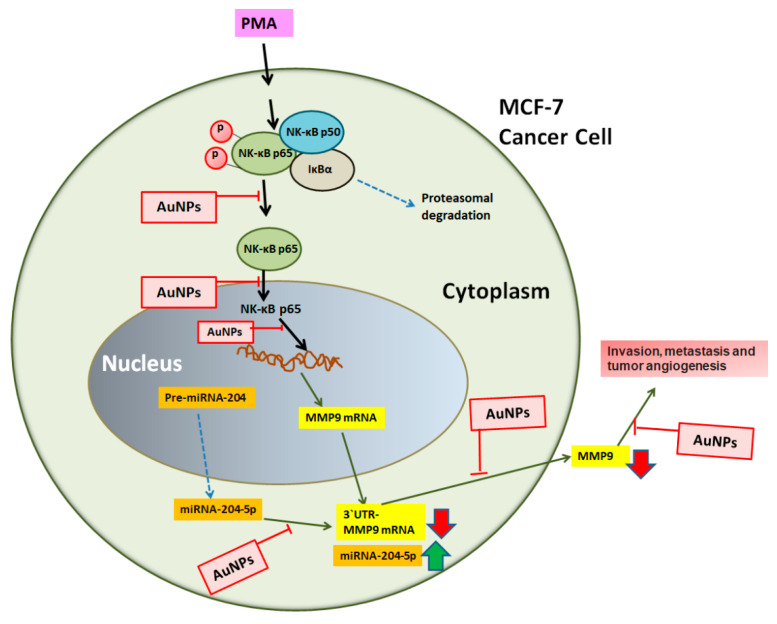
Schematic diagram of AuNPs therapeutic potential investigated in this study. PMA activates NF-κB mediated signaling to MMP-9 via microRNA-204-5p. All possible offered targets of AuNPs induced inhibition to MMP-9 are systematically shown. NF-κB complex was demonstrated by the NF-κB p65 subunit, and the pathways showing breaking arrows were not investigated. Abbreviation: AuNPs, gold nanoparticles; PMA, phorbol-12-myristate-13-acetate; NF-κB, nuclear transcription factor-kappa B; MMP-9, matrix metalloproteinase-9.

This is the first report that shows that the engineered AuNPs inhibit MMP-9 expression and production via upregulation of microRNA-204-5p in stimulated human breast cancer cells. The outcomes of this study have been schematically presented in Figure 10. Breast cancer is the main cause of cancer-related mortality among women worldwide [19]. Recently, there has been a significant improvement in the management of breast cancer, but still, nearly 40% of females with breast cancer have died [20]. Recent reports indicate that breast cancer is highly resistant to chemotherapeutic agents and radiation [21,22]. Studies showed that chemotherapeutic agents, including tamoxifen, bind to the estrogenic receptors and act as an antagonist in breast tissues but as an agonist in nearby tissues [23,24], therefore chemotherapeutic treatment for breast cancer patients is highly risky [25,26,27]. Several breast cancer cases have been categorized by the rapid invasion of breast cancer cells and metastasis at nearby sites [26,27]. Invasion and metastasis have now been well-considered essential properties of cancer cells and have been assumed to be the main causes of breast cancer-associated mortality [26,27]. Both invasion and metastasis processes involve a set of proteolytic enzymes, which play a role in the degradation of tissue barriers, including proteolytic cleavage of extracellular matrix and degradation of basement membrane [28,29]. Degradation of the extracellular matrix requires the activation of extracellular proteinases, particularly MMPs, and their role has now been well-defined in cancer tissue invasion and metastasis [30,31]. Out of all MMPs as a family, MMP-9 is extremely critical for the migration of cancerous cells via invasion and metastasis [32,33,34]. In BC, MMP-9 specifically degrades type IV collagen in the extracellular matrix while promoting the progression of tumor invasion and metastasis [32,33,34]. MicroRNAs (miRNAs), small non-coding RNA molecules, have now been well demonstrated to be involved in regulating many fundamental processes via the modulation of numerous pathogenic genes [35]. In BC, the pathogenic role of miRNAs has been well-defined, and now it is well-assumed that several miRNAs are implicated in the tumor invasion and metastasis of BC cells [36]. Importantly, recent studies also determined that attenuation of microRNA-204-5p enhances the cell growth of human choriocarcinoma, suppresses apoptosis of cancerous cells, and plays a powerful role in pregnancy-induced hypertension [37]. Moreover, the role of hsa-miR-204-5p has also been demonstrated in atherosclerosis [38]. However, the role of hsa-miR-204-5p in the regulation of pathogenic genes and their associated molecular mechanisms in breast cancer has never been investigated. This study demonstrated that microRNA-204-5p regulates the expression of MMP-9 in breast cancer cells MCF-7. To prove this, we first performed the bioinformatics algorithm. TargetScan bioinformatic analysis showed 3′UTR of human MMP-9 mRNA has a complementary matched sequence for hsa-miR-204-5p. Previous studies have shown that MMP-9 has markedly expressed in several cancers, including BC tissues and their associated cancer cells [34,39]. MMP-9 expression is modulated by various physical stimulators such as PMA, and PMA has now been well considered as one of the most common agents as a cancer initiator that enhances MMP-9 expression [40]; therefore, PMA was used in this study as a stimulating agent of BC cells. 

To validate this bioinformatics prediction, MCF-7 cells were stimulated with PMA, and the levels of microRNA-204-5p and MMP-9 mRNA were quantified by their specific TaqMan assays using real-time quantitative PCR. Stimulation of MCF-7 cells with PMA significantly downregulated the expression of hsa-miR-204-5p and significantly upregulated the mRNA expression of human MMP-9. Moreover, this effect was also observed in the concentration of MMP-9 secretion in the culture medium of these PMA-stimulated MCF-7 cells. These results depict an inverse correlation between microRNA-204-5p and MMP-9. To verify these outcomes, MCF-7 cells were transfected with anti-miR-204 and then stimulated with PMA. The data reflects that the treatment of anti-miR-204-5p-transfected MCF-7 cells with PMA significantly inhibited the expression of miR-204-5p expression compared to those sets of anti-miR-204-5p-transfected MCF-7 cells, but without PMA treatment, and the findings were similar when compared with non-transfected MCF-7 cells but stimulated PMA alone. Interestingly, these findings were completely reversed when we estimated the MMP-9 mRNA and protein concentrations. Treatment of anti-miR-204-5p-transfected MCF-7 cells with PMA significantly increased the expression of MMP-9 mRNA as compared with those sets of anti-miR-204-5p-transfected MCF-7 cells without PMA treatment and with those sets of MCF-7 cells which were non-transfected MCF-7 but stimulated PMA alone. These findings confirmed the inverse correlation between hsa-miR-204-5p and MMP-9 expression in MCF-7 cells and also validated that microRNA-204-5p is a direct regulator of MMP-9 expression. To confirm whether this inverse regulation between miR-204-5p and MMP-9 is involved in 3′UTR of MMP-9 mRNA, we used the reported luciferase assay using co-transfection of MCF-7 cells with a reporter clone containing the entire 3′UTR of MMP-9 and anti-miR-204-5p. A significant increase in luciferase activity was observed in MCF-7 cells co-transfected with MMP-9 3′UTR and anti-miR-204 compared to those MCF-7 cells transfected with MMP-9 3′UTR alone. The transfection of MCF-7 cells with MMP-9 3′UTR alone significantly reduced the relative luciferase activity compared to those MCF-7 cells transfected with empty 3′UTR. These findings confirmed the involvement of 3′UTR of human MMP-9 mRNA in the regulation of hsa-miR-204-5p activity. These findings are novel and have not been investigated before.

In recent times, nanotechnology has become an attractive field in medical science with the advancement in the development of functionally engineered gold nanoparticles (AuNPs) [41,42]. Chemically synthesized AuNPs can be applied to a wide range of medical applications and have also been demonstrated as anticancer agents [43]. In this study, the therapeutic efficacy of AuNPs was tested against the overexpression of MMP-9 in BC cells. We synthesized AuNPs by trisodium citrate methods, and engineered AuNPs were characterized by dynamic light scattering, UV-Vis spectrophotometry and TEM. The average size of AuNPs was quantified to be 28.3 nm, whereas zeta potential was measured to be −32.2 mV, and the polydispersity index of the AuNPs was determined to be 0.435. Moreover, the surface plasmon resonance peak was observed at 524 nm. The TEM analysis clearly showed that the overall shape of engineered AuNPs was spherical. All these properties clearly indicate that chemically engineered AuNPs were pretty stable and suitable for cancer cell treatment. In support of these observations, the viability of MCF-7 against chemically synthesized AuNPs was tested, and AuNPs were found to be non-toxic to MCF-7 cells. Furthermore, penetration of AuNPs into the MCF-7 cells was also studied by SEM imaging. The visualized SEM images clearly showed that engineered AuNPs easily penetrated cancer cells in a time-dependent manner. After confirming the non-toxic effects of AuNPs, their therapeutic potential was evaluated. Treatment of MCF-7 with AuNPs before the stimulation with PMA significantly inhibited the MMP-9 mRNA, as well as MMP-9 protein secretion. These findings indicate that engineered AuNPs possess anticancer potential against the PMA-induction of MMP-9.

It is well documented that microRNA-204-5p has been associated with the pathogenesis of several cancers, such as prostate cancer, hepatocellular carcinoma, colorectal cancer, gastric cancer, lung cancer and brain tumor [43], but its role has never been explored in breast cancer. Therefore, targeting hsa-miR-204-5p might be employed in the novel treatment of breast cancer therapy. In this study, we demonstrated that treatment of MCF-7 cells with AuNPs prior to the stimulation with PMA significantly upregulated the expression of miR-204-5p, and significantly inhibited MMP-9 mRNA expression and MMp-9 secretion in the culture medium. To validate the therapeutic effects of AuNPs on MMP-9 expression via hsa-miR-204-5p, the MCF-7 cells were transfected with anti-miR-204-5p and then treated with AuNPs after 72 h post-transfection. Transfection of MCF-7 cells with anti-miR-204-5p significantly downregulates the expression of hsa-miR-204-5p, whereas treatment of anti-miR-204-5p-transfected-MCF-7 cells with AuNPs, significantly upregulated the expression of miR-204-5p. Interestingly, this condition was reversed when we estimated the levels of MMP-9 mRNA and MMP-9 secretion in the same sets of anti-miR-204-5p-transfected MCF-7. These findings indicate that AuNPs inhibit MMP-9 expression/production via upregulation of hsa-miR-204-5p expression. To confirm our central assumption that inhibition of MMP-9 expression by AuNPs via involvement of 3′UTR of MMP-9 mRNA and hsa-miR-204-5p. This assumption was validated by a luciferase reporter assay. Our findings showed a significant increase in the relative luciferase activity in MCF-7 cells co-transfected with MMP-9 3′UTR reporter and anti-miR-204-5p. This increase in luciferase activity was inhibited by the treatment of these co-transfected MCF-7 cells with AuNPs. These findings clearly indicated that AuNPs inhibit MMP-9 expression via upregulation of hsa-miR-204-5p through 3′UTR of MMP-9 mRNA in MCF-7 cells. The therapeutic potential of AuNPs against MMP-9 expression via hsa-miR-204-5p was further re-evaluated by transfection with anti-miR-204-5p and then treated with PMA and AuNPs. Treatment of anti-miR-204-transfected MCF-7 cells with PMA significantly inhibited the expression of hsa-miR-204-5p compared to non-transfected MCF-7 cells stimulated with PMA. Interestingly, treatment with AuNPs significantly upregulated PMA-inhibited hsa-miR-204-5p expression. This condition was reversed when we estimated MMP-9 mRNA and MMP-9 protein secretion. Stimulation of anti-miR-204-5p-transfected MCF-7 cells with PMA significantly enhanced the expression of MMP-9 mRNA as compared with non-transfected MCF-7 cells stimulated with PMA. Interestingly, treatment with AuNPs significantly inhibited PMA-induced MMP-9 mRNA expression. To determine whether this inhibition of MMP-9 mRNA expression also affected the protein level, the cell culture medium of these transfected or non-transfected MCF-7 cells was analyzed. The data showed that treatment with AuNPs significantly inhibited PMA-induced MMP-9 protein secretion. Furthermore, these findings were further re-validated by gelatin zymography based MMP-9 activity in the culture medium of anti-miR-204-5p-transfected MCF-7 cells treated with AuNPs and PMA. Anti-miR-204-5p transfected MCF-7 cells were pretreated with AuNPs for 2 h before the stimulated by PMA for 24 h, and equal volumes of culture medium were loaded on a polyacrylamide gel and MMP-9 activity was analyzed by gelatin zymography. These findings clearly indicated that treatment with AuNPs inhibited PMA-induced MMP-9 activity. The data explicitly demonstrates that AuNPs inhibited MMP-9 mRNA and MMP-9 secretion via upregulation of xhsa-miR-204-5p expression.

NF-κB is a master transcription factor for the induction of numerous proinflammatory signaling events; its activation is directly associated with multiple pathogenic processes in cancer [44]. Activation of NF-κB signaling via movement of its subunit p65 in the nucleus has now been considered one of the main pathogenic mechanisms involved in tumorigenesis and has now been well-considered to be the best target for cancer therapy [45]. Importantly, studies reported that stimulation of cancer cells with PMA induces MMP-9 expression via activation of NF-κB signaling pathway [40]. In this study, we determined that AuNPs inhibit PMA-induced NF-κBp65 activation via the involvement of MMP-9 and hsa-miR-204-5p. Our findings showed a significant increase of NF-κBp65 levels in the nuclear extract of MCF-7 cells treated with PMA alone for 30 min, and the response was also similar in MCF-7 cells transfected with anti-miR-204-5p. Interestingly, a sharp increase in the levels of NF-κBp65 was observed when transfected MCF-7 cells with anti-mR-204-5p were stimulated with PMA, and this increase of NF-κBp65 was significantly inhibited by AuNPs. To confirm that our experimental setup was suitable for the determination of NF-κB activity, we treated anti-miR-204-5p-transfected MCF-7 cells with parthenolide, a well-known marker for the determination of NF-κB inhibition [46,47]. Treatment of anti-miR-204-5p-transfected MCF-7 cells with parthenolide showed significant inhibition of PMA-induced MMP-9 mRNA, MMP-9 protein secretion and NF-κBp65 activation. Interestingly, the expression of hsa-miR-204-5p was significantly upregulated in the same set of transfected MCF-7 cells treated with parthenolide. These findings confirmed the involvement of NF-κBp65 inhibition by parthenolide or AuNPs via modulation of hsa-miR-204-5p and MMP-9 expression. Altogether, these findings confirmed that the engineered AuNPs inhibit MMP-9 expression via hsa-miR204-5p upregulation and NF-κBp65 deactivation in human breast cancer cells. 

## 5. Conclusions

This is the first report which demonstrates that newly engineered AuNPs inhibit PMA-induced MMP-9 gene expression and protein production, leading to the deactivation of NF-κBp65. The progress of the signaling events occurs via the upregulation of microRNA-204-5p expression in human breast cancer cells. These novel therapeutic actions of engineered AuNPs on stimulated breast cancer cells provide novel suggestions that AuNPs inhibit tumor-promoting activity via the modulation of microRNAs.

## Figures and Tables

**Table 1:** Characterization of AuNPs prepared by trisodium citrate.

Technique	Parameter	Findings
Dynamic Light Scattering	Average Size	28.3 nm
	Zeta Potential	−32.2 mV
	Polydispersity Index	0.435
UV-Vis Spectroscopy	Surface Plasmon Resonance Peak	524 nm
Transmission Electron Microscopy	Shape	Spherical

## Data Availability

All data and materials used in this study are available with the corresponding author and will be provided upon reasonable request.
